# Alcohol Availability, Marketing, and Sexual Health Risk Amongst Urban and Rural Youth in South Africa

**DOI:** 10.1007/s10461-018-2250-y

**Published:** 2018-08-21

**Authors:** Lebohang Letsela, Renay Weiner, Mitzy Gafos, Katherine Fritz

**Affiliations:** 1 Soul City Institute for Social Justice (SCI), 281 Jan Smuts Ave, Cnr Bompas Rd, 1st Floor Dunkeld West Centre, Dunkeld West, Johannesburg, South Africa; 2 School of Public Health, University of the Witwatersrand, York Road, Parktown, Johannesburg, South Africa; 3 London School of Hygiene & Tropical Medicine (LSHTM), Room 328, 15-17 Tavistock Place, London, WC1H 9SN UK; 4 International Center for Research on Women (ICRW), 1120 20th St. NW, Suite 500N, Washington, DC 20036 USA

**Keywords:** Youth, Photovoice, GIS mapping, Alcohol marketing, HIV

## Abstract

South Africa has high rates of HIV prevalence and incidence and very high binge drinking rates amongst youth. GIS mapping of alcohol outlets and participatory research methods were used to explore and understand youth’s perception of alcohol availability and marketing, and their associated risks for HIV and sexual violence. Twenty seven youth 18–24 years from an urban and rural area participated in the study. There was high density of alcohol outlets at both sites with 97% within 500 m radius to a school; 76% of outlets had alcohol advertising signage on the exterior. Youth reported that alcohol is easily accessible to them. Alcohol advertising and marketing are appealing and entice them to consume alcohol, including binge drinking. Young women reported exposure to unsafe and transactional sex, and sexual violence at alcohol outlets. Effective policies are needed to regulate alcohol availability and marketing to youth.

## Introduction

High HIV prevalence and high levels of alcohol misuse continue to be major public health concerns in South Africa, with youth experiencing particular vulnerability to both [1, 2]. South Africa continues to have the highest burden of HIV infection worldwide [3, 4], with an estimated one-fifth of women of reproductive age infected [5].

Youth drinking between the ages of 11–20 years in South Africa is of concern as it is estimated that 32% of youth, with more males (36.6%) than females (28%), had drunk alcohol in the past month [6, 7]. The 2013 Youth Risk Behaviour Survey (YRBS) revealed that 12% (16% males and 8.7% females) of teenagers initiate alcohol use before 13 years of age. One quarter of youth reported binge drinking with significantly more males (30%) than females (20%) engaging in harmful drinking in the past month. This is higher than youth rates in other parts of the world, for example, 21.9% of youth (23.8% males and 19.8% females) in the USA reported binge drinking in the past month [6, 7]. Binge drinking was defined as consumption of ≥ 5 alcoholic beverages within a few hours on one or more days in the preceding month [8].

Numerous cross-sectional and longitudinal studies conducted over the past 20 years have shown a strong association between alcohol consumption and sexual risk behavior that can lead to HIV [9]. Specifically, alcohol misuse in terms of both frequency and quantities consumed is associated with increased number of sexual partners, unprotected sex and sexual encounters that are later regretted [10–12].

Moreover, increased HIV risk has been documented amongst high-risk alcohol drinkers 15 years and older [11]. In a review of 50 studies from Sub-Saharan Africa, Morojele and colleagues found that adolescents who drink are more likely to have early sexual debut and engage in multiple sexual partnerships [13]. Eighteen percent (18%) of youth reported having sex after consuming alcohol (20% males and 13.9% females) [7]. Numerous research studies have established that bars, including taverns, restaurants and shebeens are places where heavy alcohol consumption takes place along with associated HIV sexual risk behaviors [14–18].

The ubiquitous availability of inexpensive alcohol and its promotion through advertising and sponsorship by the alcohol industry have been increasingly recognized as a driver of alcohol misuse and thus ill health. The World Health Organization (WHO) has led global efforts to reduce the harmful use of alcohol and developed a strategy to this end [19]. The strategy recommends evidence-based approaches to reducing alcohol-related harm, among which is the need for national alcohol policies to be guided by public health priorities rather than by the interests of the alcohol industry. The WHO global alcohol strategy recommends restricting alcohol availability to youth through establishing a minimum age of consumption, limiting the number and location of alcohol outlets and reducing the impact of appealing marketing to which youth are exposed. It also recommends that children, teenagers and adults be protected from harmful drinking norms that pressure them to drink alcohol. These policy approaches have not been adopted in South Africa, where addressing alcohol as part of a comprehensive HIV-prevention strategy for youth is both complex and crucial. The Control of Marketing of Alcoholic Beverages Bill was drafted in 2010 by the National Department of Health to reduce the effects of alcohol marketing in advertising and promoting alcohol use and consequent harm. The bill aimed to contribute to the reduction of alcohol-related harm and the protection of public health and community well-being by limiting exposure to alcohol marketing through restricting the advertisement of alcoholic beverages, prohibiting any sponsorship associated with alcoholic beverages and prohibiting any promotion of alcohol beverages [20]. This bill has not yet been passed into a law and raised much controversy, particularly from the alcohol and advertising sectors, who argue that banning alcohol adverts would have a negative economic impact, including significant job losses [20].

The evidence supporting the harmful effects of alcohol advertising on youth is compelling. A systematic review of eight cohort studies that followed a total of 13,000 youth aged between 10 and 26 years from developed countries, demonstrated that alcohol advertising was associated with initiation of drinking and hence a substantial public health risk to youth [21]. Furthermore, a recent study that was conducted by Naimi et al. with 1032 teenagers aged between 13 and 20 years in the US further confirmed the relationship between exposure to alcohol brand advertising and brand specific consumption in the past 30 days [22, 23].

Globally, there is little published qualitative data that explores the pathways through which alcohol advertising and drinking interact among young people. We know of no South African study that has documented young people’s perspectives on alcohol availability and marketing, and their influence on drinking patterns and sexual health including HIV risk behavior.

The purpose of this study was to address these gaps and to offer a community and youth-based perspective of harmful alcohol use, sexual risk and factors that drive these. Despite much national level alcohol policy debate about WHO’s recommended legislation, policy debate in South Africa has taken place in the absence of community voices and their views of alcohol, its consequences and the role of alcohol advertising.

The study conducted by the Soul City Institute is part of the STRIVE research consortium led from the London School of Hygiene and Tropical Medicine (LSHTM), with partners in India, Tanzania, and South Africa. STRIVE investigates the structural forces—in particular stigma, gender inequality and violence, and alcohol availability and drinking norms—that combine in various ways to shape vulnerability to HIV transmission and undermine prevention efforts.

The aim of the study was to examine the drivers of young people’s alcohol consumption and related vulnerability to HIV and sexual violence. More specifically, the study intended to:

1. Document the availability of alcohol in the target communities, in terms of density of alcohol outlets, pricings and promotions.
2. Explore relationships between the availability, promotion and pricing of alcohol and young people’s drinking norms, drinking patterns, sexual risk behavior and experiences of sexual violence.
3. Based on objectives 1 and 2, identify opportunities and make recommendations for changing alcohol policies to better protect young people from alcohol-related sexual risk.

## Methods

### Setting

Two South African communities were selected to contrast how alcohol affects youth in contexts that have different resources, infrastructure and access to services. An urban township, situated in the Tshwane district in the Gauteng Province and a rural community in the Nkangala district in Mpumalanga Province were chosen.

The urban area has a population size of 60,425 people. Sixteen percent (15.8%) of the population over 20 years of age have graduated with a higher education qualification while 4.5% of this population has no schooling. Moreover, 42.9% of households are female headed [24]. The unemployment rate in the district within which the community is based is 28.3% (29.2% in Gauteng Province) compared to the national rate of 27.7% [25]. The urban area has strong infrastructure with tarred roads connected through regional roads. Ninety-three percent (93%) of the population reside in formal housing; 98% of households have electricity for lighting; 67% have piped water and 99% use a flush toilet connected to a sewerage system [24].

Mpumalanga where the rural community is based is in the eastern part of the country approximately 180 km from Johannesburg. It has a population size of 20,793 with 2.9% of adults 20 years and older possessing a higher education qualification, while 20.4% of this population has no schooling. Furthermore, 49.3% of households are female headed [25]. The unemployment rate in the Mpumalanga Province is 31.5% (no data is available for the district) [25]. The infrastructure is poor with roads being mostly untarred, connecting to the main national road. While 81% of households reside in formal housing and 95% have electricity for lighting, only 8.4% have piped water inside their dwelling and 80% use a pit toilet system [24].

### Data Collection

Two data collection methods were used: Geographic Information System (GIS) mapping and Photovoice.

#### GIS Mapping

Observational data on the number and location of all alcohol licensed outlets and schools in the community were collected using a standard template and a handheld Global Positioning System (GPS). A researcher and locally recruited fieldworker traversed every street in communties within perimeters defined on Google Maps, using a motor vehicle and stopping in front of outlets and advertisements, and recorded the GPS coordinates of each outlet that could be observed to sell or serve alcohol as well as alcohol advertisement posters or billboards. The mapping process took 3 days in the rural site and 5 days in the urban site. The mapping excluded hidden (usually unlicensed) alcohol selling places. For each alcohol serving or selling establishment, a data entry form was completed to record the type of venue, signage of operating hours and whether a minimum legal age of drinking alcohol and any advertising and pricing was posted on the exterior of the building. The GPS coordinates were downloaded from handheld GPS trackers onto the computer and data points were transferred to the open source Quantum Geographic Information System (QGIS).

#### Photovoice

Photovoice is a participatory action research method that engages participants in documenting their world using photographic images that are interpreted with captions [26, 27]. The photographic images are complemented with focus group discussions (FGDs), called photo dialogues. This methodology was used because of its participatory nature and innovation in employing photography to identify and represent phenomena. It was deemed an exciting and interactive approach to use with youth as opposed to traditional FGDs to explore their perceptions of exposure to alcohol advertising, drinking patterns and consequences of alcohol consumption in their communities. A workshop manual was developed to guide the sessions and comprised learnings, practical exercises, photo dialogues and focus group discussions. All 27 youth participants (13 and 14 in the rural and urban areas, respectively) participated in all the photovoice sessions and activities. A series of six workshops lasting 4.5 hours a day were held twice a week over a period of 3 weeks at each site.

The workshops covered basic photography techniques such as how to use a camera, basic principles and techniques of photography (for example, framing, focus, follow through and flash), ethical issues and model consent in taking photos, camera safety, visual literacy and text to describe their photos (captioning), how to use symbolism in order to express ideas and feelings, and dialogues on benefits and consequences of alcohol consumption also using existing photos. Youth participants (n = 27) did homework exercises that were used in photo dialogues in the sessions. These included taking photos of all alcohol adverts they were exposed to in their daily lives, capturing images of their perceptions and experiences of how alcohol affects them and their communities. In the later sessions participants also did scrapbooking and captioning using their own photos taken during their homework and in session exercises. Each participant took an average of 18 photos and chose their top 10 photos and corresponding captions that were printed and displayed at the celebration event which was attended by participants’ family and friends at the last session.

Convenience sampling was used to select youth to participate in the study. Both in and out of school youth, aged 18–24 years were recruited to participate through the Soul City *Rise* young women’s club program and local youth Community Based Organisations (CBOs). *Rise* young women clubs aim to prevent HIV among young women and girls (YWGs) aged 15–24 years through tackling structural drivers of HIV such as gender-based violence, gender inequality and poverty. The program is implemented in five provinces with a membership of a maximum of 20 YWGs in each club. Clubs hold regular meetings and conduct projects dealing with HIV prevention. The clubs are complemented by the Rise TV Talk Show that was broadcast on a national television channel, SABC 1. The young men were recruited through local CBOs who referred young men participating in their youth programs, particularly computer literacy and lifeskills or health awareness programs.

Two researchers facilitated the photovoice workshops including eight focus group discussions at each site. A structured discussion guide was used to facilitate four sex-segregated focus group discussions that explored the benefits, risks and consequences of alcohol, including how alcohol affects “you and the community” during sessions 3 and 5, and four mixed group discussions with the same participants explored the role of alcohol advertising, promotions and pricing on youth alcohol intake during sessions 2, 4 and 5. The latter FGDs also used the photos taken by the youth during their homework exercises after session 3 and 4 to generate more discussion about youth exposure to alcohol marketing, and their drinking habits and sexual behaviors as well as consequences and benefits of alcohol. FGDs were conducted in the local languages, isiZulu and isiNdebele in the rural community and Setswana in the urban township.

Research assistants were recruited from local CBOs to recruit participants, facilitate logistics, download photographs and conduct support visits between workshop sessions.

#### A Desktop Review

A desktop review of the South African alcohol legislature was conducted to gain an understanding of the policy context and identify opportunities to use the research to influence alcohol policies in order to protect young people from harmful alcohol use and related sexual risk. The review consisted of three documents:

- The Control of Marketing of Alcoholic Beverages Bill drafted in 2010.
- The South African 2003 Liquor Act.
- The national liquor norms and standards, 2015.

### Data Analysis

#### GIS Mapping

The mapping data were analyzed in QGIS and OpenStreetMaps to visualize the density of alcohol establishments and their proximity to schools. A 500 m radius was measured using a buffer on QGIS around each school to assess if outlets were located within this range. An estimate of the surface area was also calculated for each community studied. The ratio of outlet per population and outlet density were calculated for each site.

#### Photovoice: Focus Group and Photo Dialogue Discussions

Qualitative data from the group discussions (photo dialogues and guided FGDs) were recorded, translated into English and transcribed verbatim. The text documents were then imported onto the ATLAS-ti software to create Hermeneutic Units. An inductive approach (bottom- up) was used to openly create codes of text “quotations” with similar information or meaning. Related codes were then organized into groups of families using networks to express relationships between the codes and families, which emerged into themes [28, 29]. Participants were assisted with editing their photo captions to describe their photos. Photographs were primarily used for interpretation and to guide group discussions - no further analysis of these photos was done.

### Ethics

The study received ethical clearance from the South African Human Science Research Council (HSRC) ethics review board. Written informed consent was obtained from all participants. Photovoice workshops included training participants on how to obtain consent for taking and using photographs from models who were people in the community such as their friends, family as well as strangers. Identifiers were removed from all FGD transcripts so that the data were anonymized. Anonymity of the alcohol outlets was also maintained through the removal of identifiers (name of community and outlet) on the QGIS maps. Participants were given the option to use an alternative name or a pseudonym instead of their own name to identify the photographer and ascribe copyright to their photographs. This enabled them to remain anonymous when the photographs were displayed.

## Results

### GIS Mapping: Alcohol Outlet Density

#### Urban Site

**Fig. 1:**
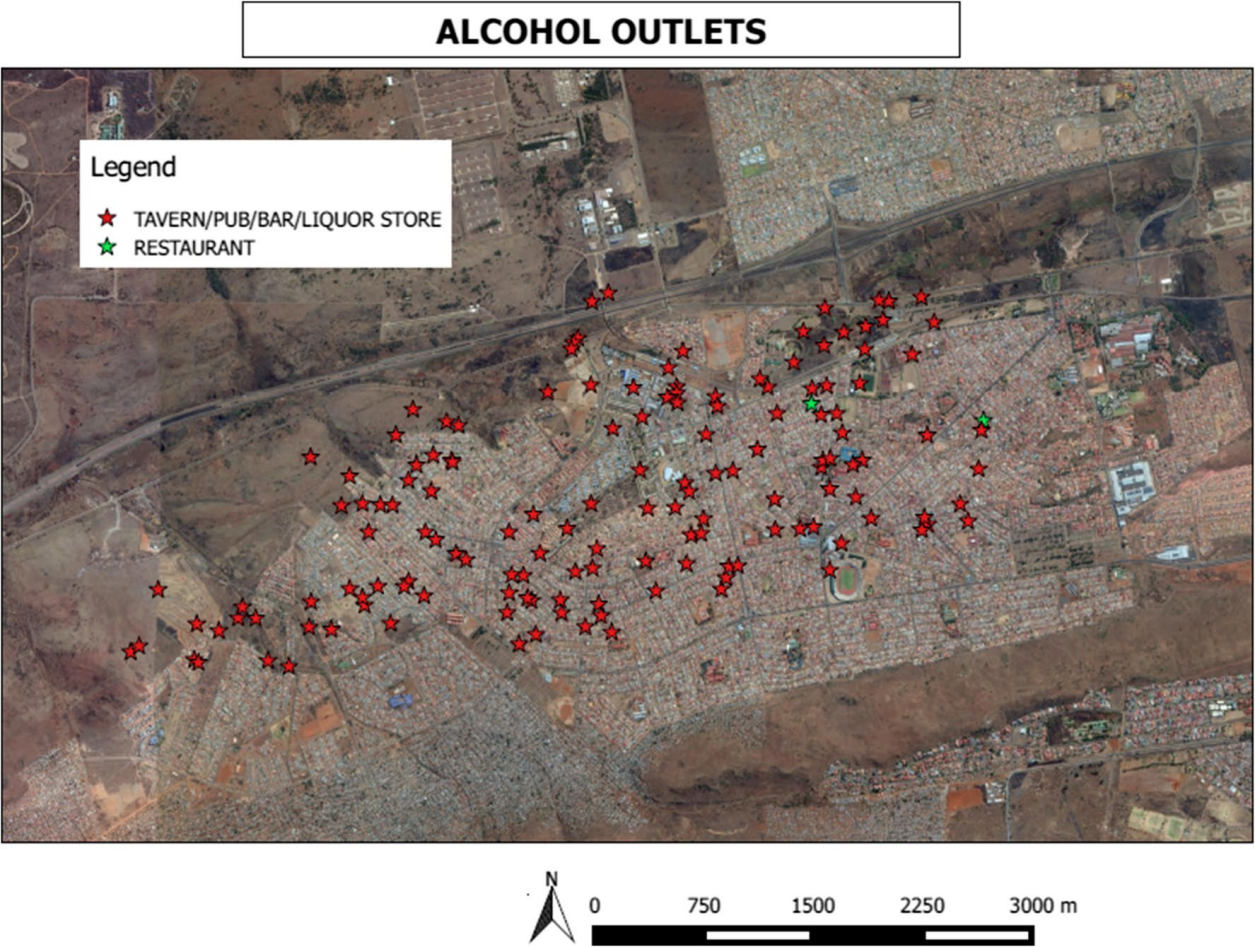
Density of alcohol outlets, urban site

The size of the urban site was estimated at 13.7 square kilometer (km^2^). A total of 147 licensed alcohol selling outlets were mapped within this area (see Fig. 1). Sixty five percent (65%) of the outlets (n = 95 ) were taverns/pubs or bars with the bulk of the remainder being liquor stores (n = 50) and two restaurants. Thus a total of 10.7 outlets were mapped per square kilometer. The ratio of an alcohol outlet to population was 1:438 with between 2 and 5 outlets per main road.

Twenty-four percent of the outlets did not have a sign for the legal age for alcohol consumption while only 3% posted their operating hours on the exterior of the buildings. Seventy-six percent (76%) had some form of advertisement outside of the venue. There were 15 stand-alone billboards and posters in the streets.

**Fig. 2:**
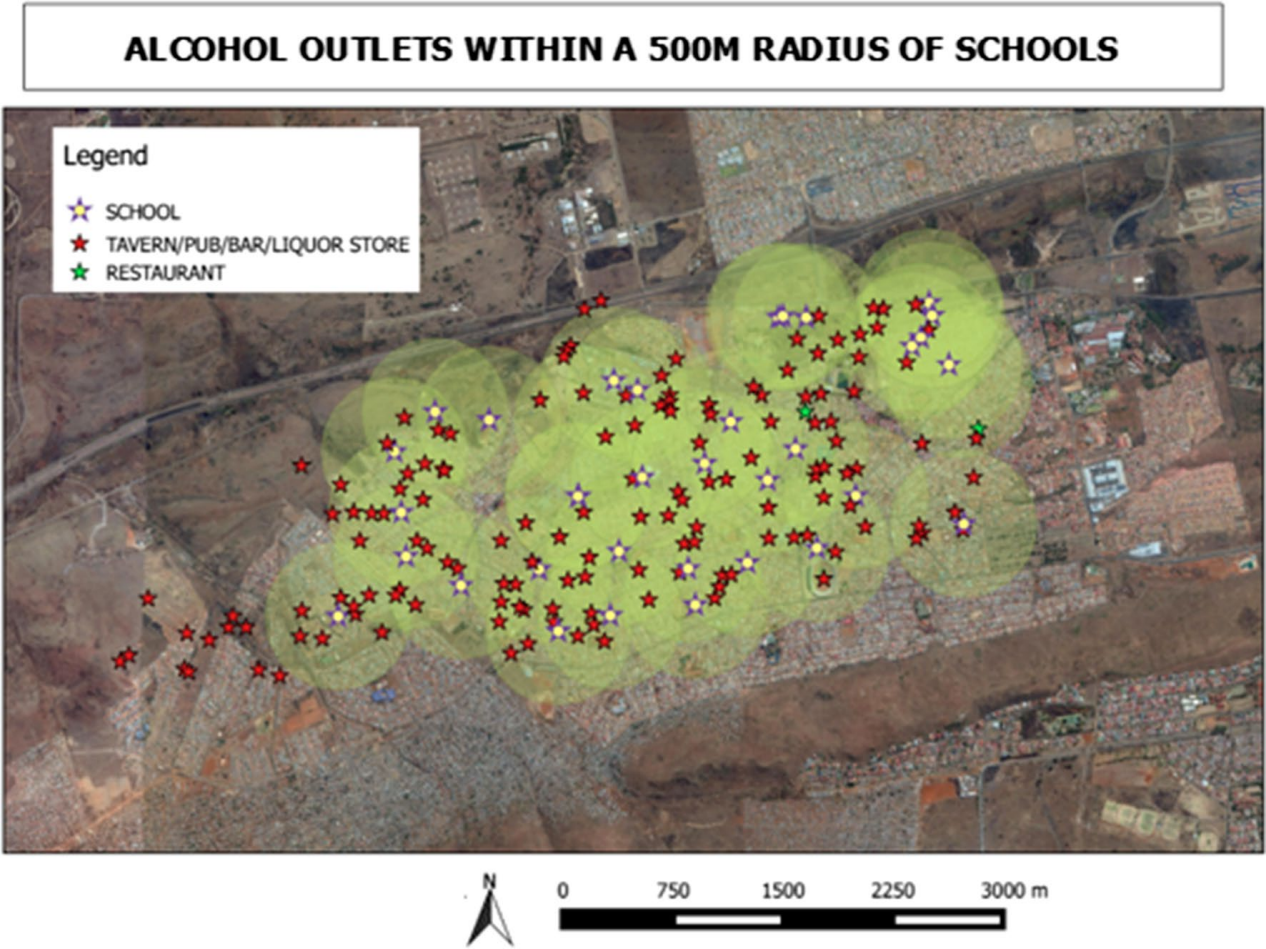
Density of alcohol outlets and educational facilities, urban site

A total of 74 educational venues were mapped. These comprised 21 primary schools, 11 senior/secondary schools, one Further Education and Training College (Technical Training), two public libraries and 39 day care centers. Figure 2 presents a 500 m radius around each school since this is the proposed legal distance allowed for outlets to be situated away from educational institutions. This shows that almost all alcohol selling outlets are within a 500 m radius to schools—the minimum distance was 10 m.

#### Rural Site

**Fig. 3:**
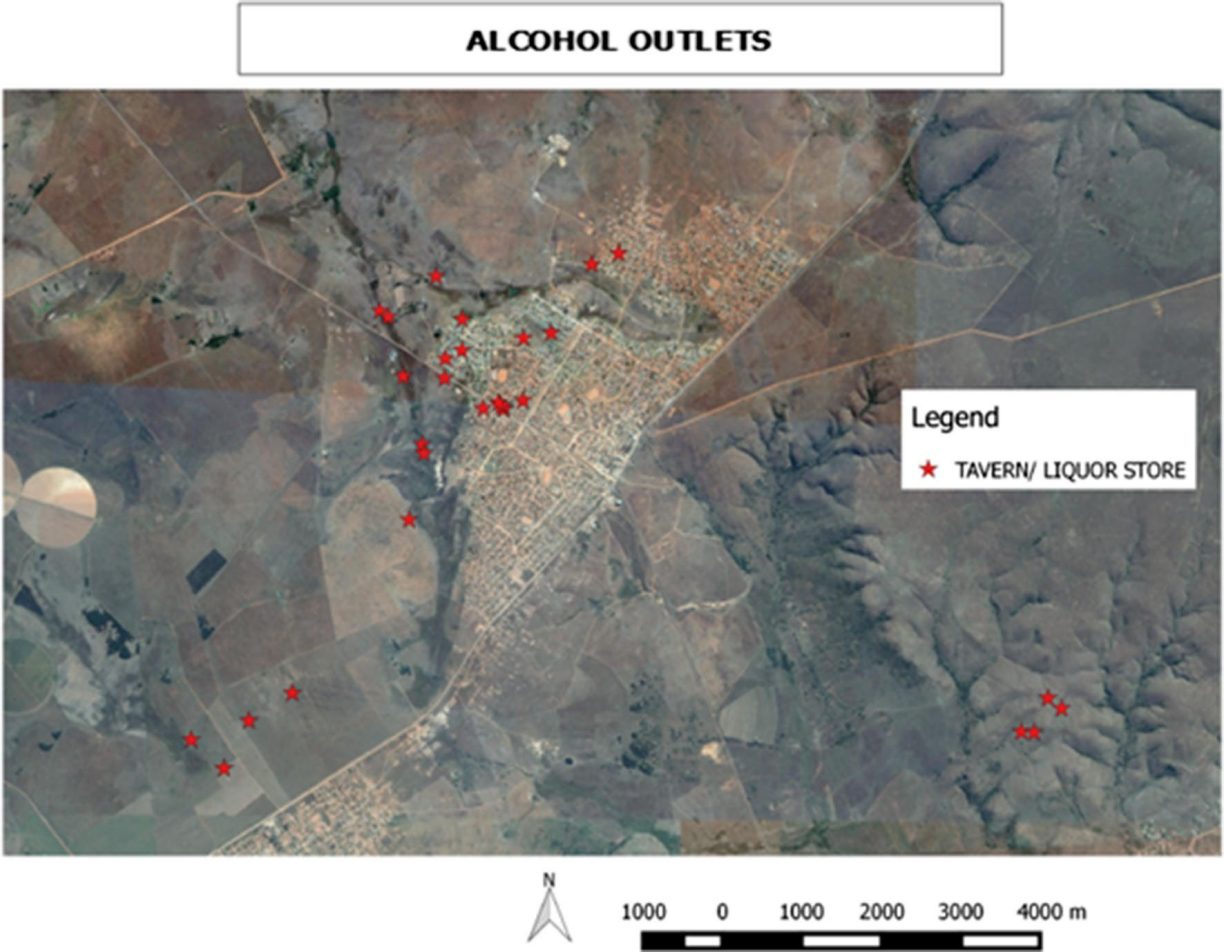
Density of alcohol outlets, rural site

The rural site total area was estimated at 17.6 km^2^. A total of 28 licensed alcohol selling outlets, 25 (89%) taverns and three liquors stores were mapped (see Fig. 3). There were 1.5 outlets per square kilometer. The ratio of an alcohol outlet to population was 1:743 with at least one outlet per tarred main road. A quarter of the outlets had a sign for the legal age while none had a sign with operating hours on the exterior of the buildings. Ninety-two percent (92%) had some form of alcohol advertisement outside of the venue. No stand-alone billboards and posters were found in the rural site.

**Fig. 4:**
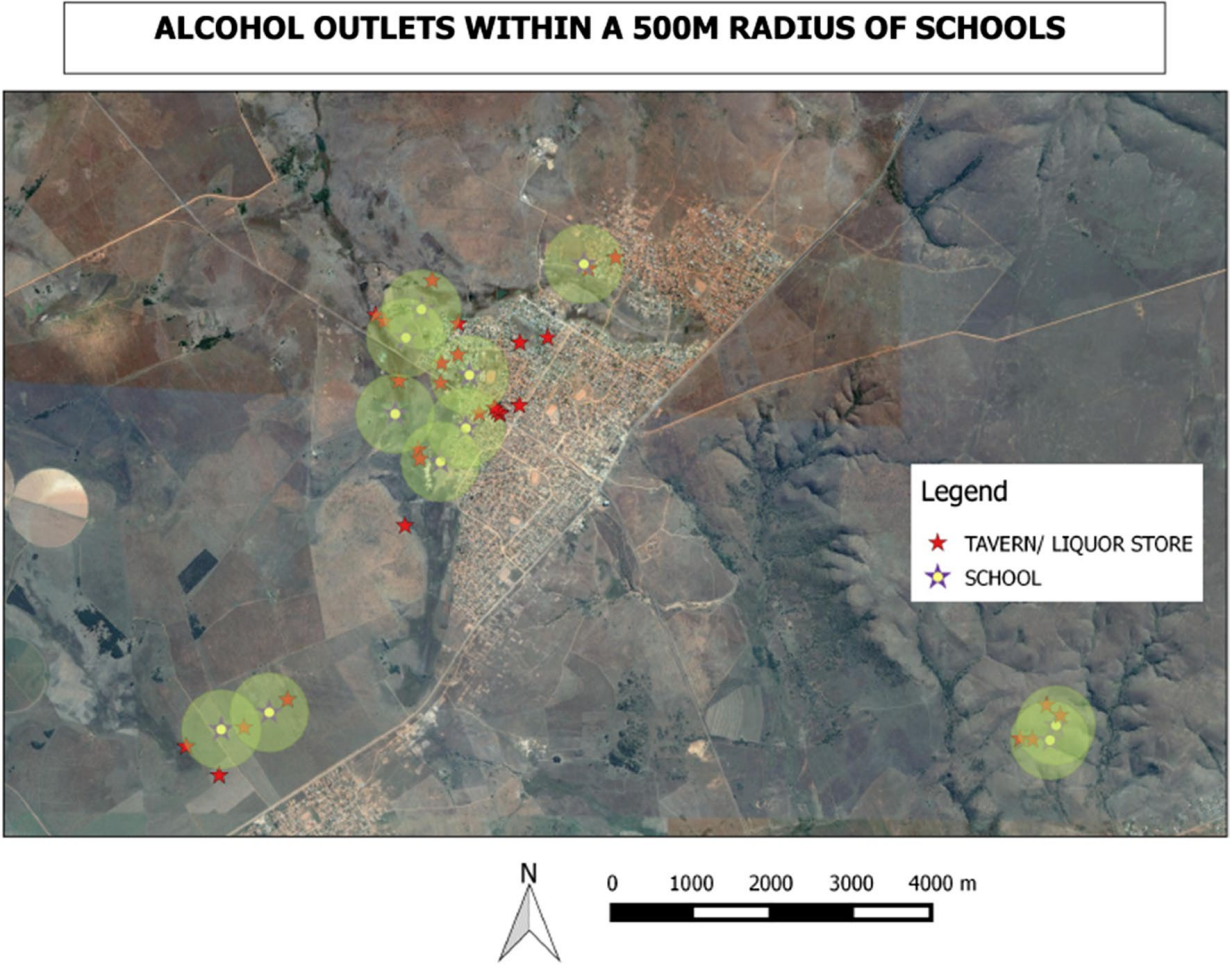
Density of alcohol outlets and educational facilities, rural site

A total of 11 schools were mapped—six primary and five secondary. No libraries could be found during the mapping exercise. Figure 4 indicates that 22 of the 28 outlets (79%) were within a 500 m radius to schools, closer than the legal distance. The minimum distance was 100 m and the maximum 700 m. Thirty-two percent (32%) of the alcohol-selling outlets sold groceries or had a grocery shop on the same premises, thus facilitating easy access of alcohol during school breaks.

### Photovoice

A total of 27 youth participated in all the photovoice activities; 13 (six females and seven males) in the rural area and 14 (six females and eight males) in the urban area.

#### Alcohol Availability

**Fig. 5:**
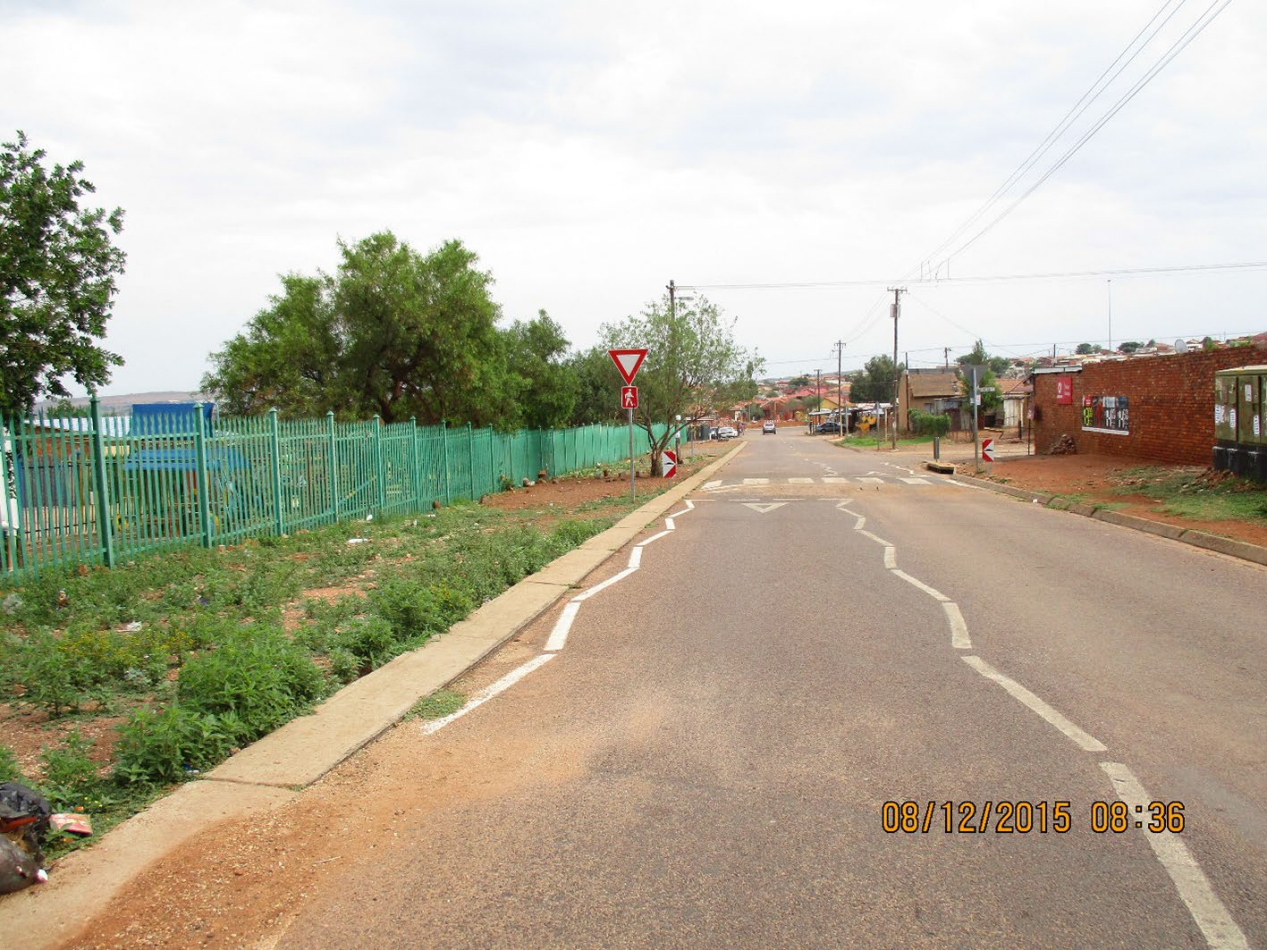
Alcohol is so available in our communities. Look at this tavern on a main road, and right opposite a school! © Fifi/11-12-15/Soul City Institute. *Source:* Photo taken by a photovoice participant—female, 19 years old, Urban Site

The mapping findings were supported by the FGD data, with participants indicating that taverns and schools co-existed in close proximity. Figure 5 is a photograph of the proximity taken by youth in the urban site. According to the youth, underage drinking is widespread, including during school hours. In both communities, youth reported that alcohol was widely available to them through easy access to taverns where age verification checks were absent and youth below age 18 years were easily able to purchase alcohol.

Even though the rule is ‘not for sale for persons under 18’ but still if you can look around, like go around the taverns at night or like uhm… daylight… Not everybody is above the age of 18. These people [traders] they care about money not by how our lives get ruined by alcohol [All agree] (**Male**, mixed gender group, rural site).

There are schools and taverns like in the same street … the fact is we are exposed to it like in our daily lives (**Female**, mixed gender group, urban site).

Youth reported that it is easy to sneak alcohol onto school premises as there is no monitoring or checking of students as they enter the school premises. Alcohol sellers make little effort to dissuade underage purchase of alcohol. Youth spoke about purchasing alcohol during break time since it was sold at the same shop where they bought their lunch.

[They buy at] taverns obviously and most of the taverns are near the schools so it’s easy, even during break time (lunch) it’s easy for me to get alcohol… at the taverns! And [the students] hide it… (**Male**, mixed gender group, rural site).

#### The Influence of Alcohol Pricing on Youth Drinking Behavior

Alcohol is affordable to youth even if unemployed or with little available cash. This is enabled by discounts, such as the ubiquitous “Ladies’ Night” which happen at outlets regularly:

**Female**: “They say Ladies’ Night because ladies get their alcohol for free. **Facilitator:** And then the guys buy their own…? **Female**: Yes, hence they call it Ladies’ Night! **Facilitator:** And do you get a lot of girls there in attendance? **Female**: Yes… Yho, the whole community! Even if you are 14 years, as long as you’re a girl you get in and get your free drink.” (Mixed gender group, rural site).

**Fig. 6:**
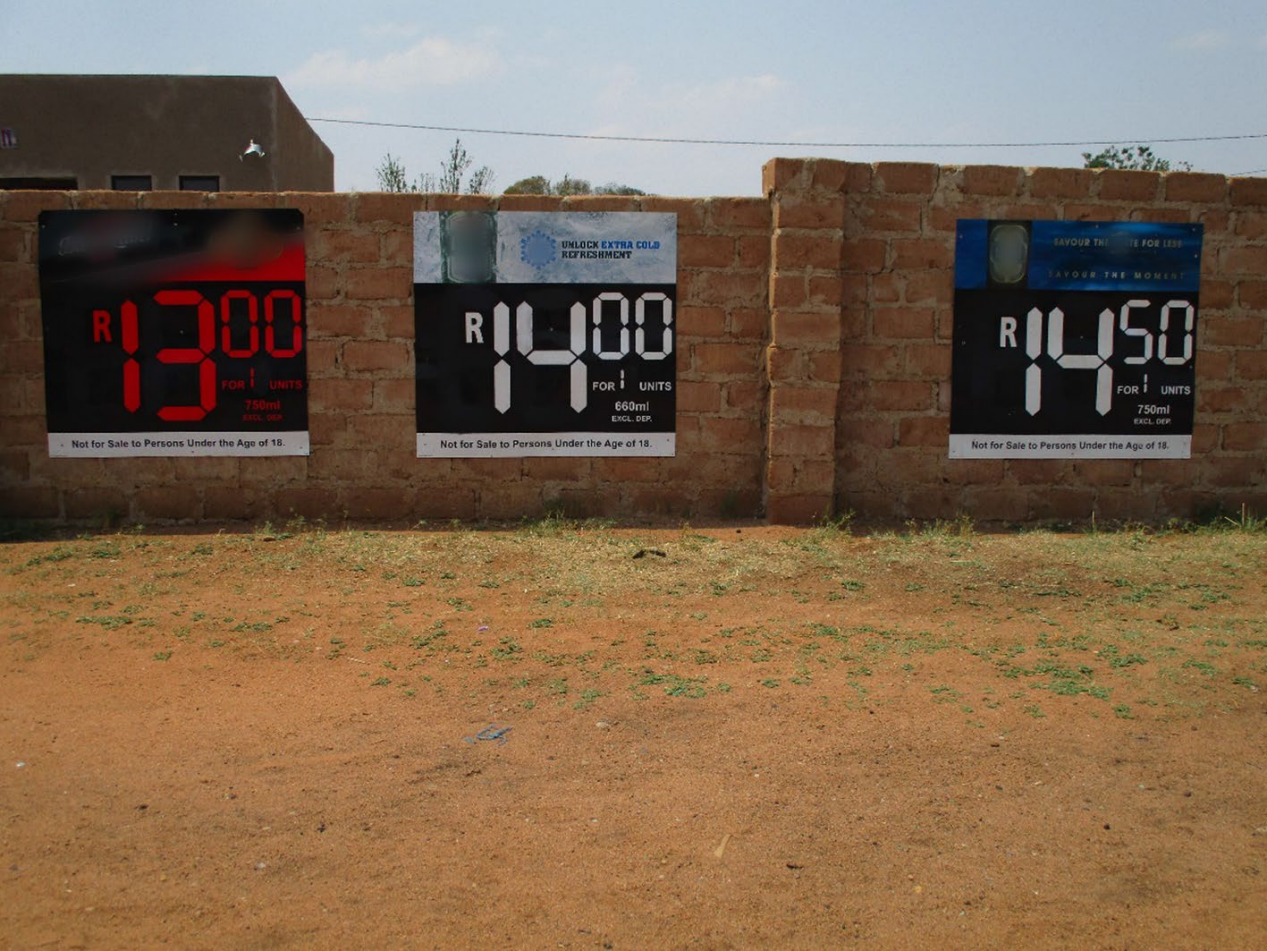
The beauty of the wall next door: as I gaze my eyes on that colorful beautiful wall I swear even those prices do not make any difference on my choice, only the colors that suits my personality will keep me going © Joel/11-12-15/Soul City Institute. *Source:* Photo taken by a photovoice participant—male, 21 years old, Rural Site

Affordability is also enhanced by incentives and competitions where the more you buy the advertised product, the more you stand a chance to win various prizes, discounts and specials such as ‘return and save’ where bottles returned lead to a discount for the next drink. This perception of saving encourages purchases of the alcohol. Figure 6 shows a photograph taken by a young person in the rural site.

**Female:** “…Do you see there on top…it shows you that you bring back the bottle and maybe they give you back R3?… Yes, you get R3 and you may have spent only R10! **Facilitator:** Aah, so looking at this as a young person, would you want to buy it? **Female:** It is not our stuff but that return for deposit is pretty impressive… **Male**: You would [want to buy it].” (Mixed gender group, urban site).

#### The Influence of Alcohol Marketing on Youth Drinking Behavior

Youth are exposed to multiple forms of alcohol marketing. These include sports sponsorship, television, social media, outdoor advertising (billboards and posters), competitions and promotional events such as comedy and music shows.

The advertisements on the billboards that we see around when maybe you are travelling… It’s the thing that attracts us to alcohol… it’s like uhm… they are not showing the negative effects. And if you watch Generations on TV for an example, when it goes to an advert, maybe 3 or 4 of those adverts, it’s alcohol… (**Male**, mixed gender group, rural site).

**Female 1:** So there are Facebook pages where there are competitions for example you would win a phone or what what. So you become eager and drink the [brand name removed] to a point of being so drunk so you can win that phone… **Female 2:** Others they share on the Facebook. The thing is you will get a notification of some promotion. Without your knowledge of how or why, you just get a notification that it [promotional event] is happening at such and such a place. So [even] if you do not follow their page then they will notify you of what what event!” (Female group, urban site).

**Female 1**: “Yes, we find them on the newspapers, magazines or so but here … is a rural area and not everybody can afford a newspaper or a magazine but most households have television. So, that’s where we get most of the information. **Female 2:** Yes, yes!! **Female 3**: Even on social media… they say on Facebook and Twitter! **Male**: Instagram…Twitter. **Female 3**: Facebook, Instagram, Twitter.” (Mixed gender group, rural site).

**Fig. 7:**
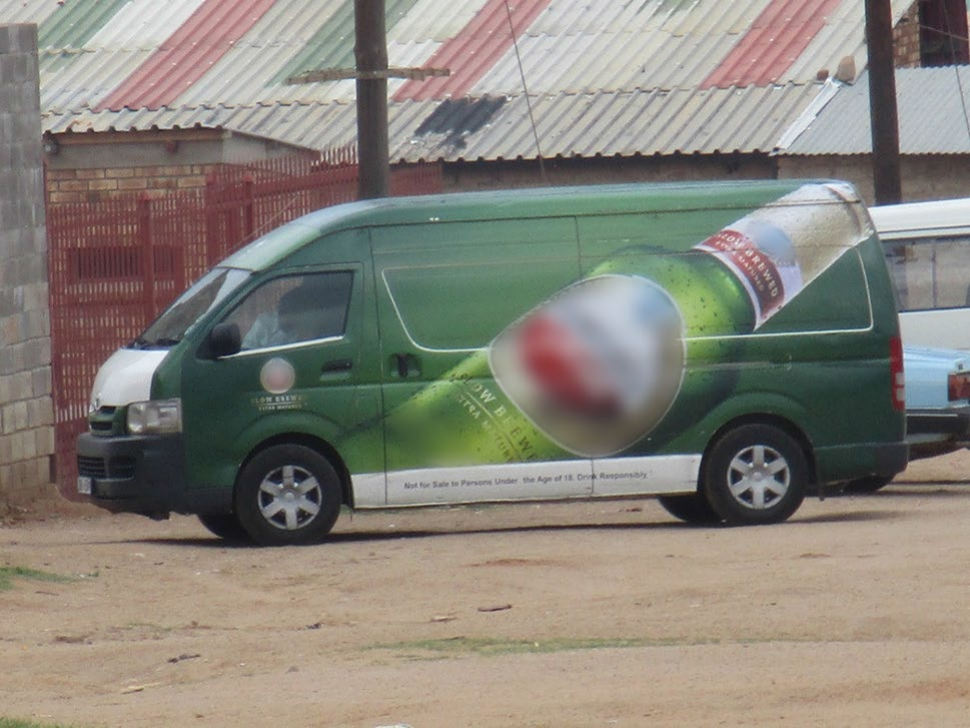
Just by looking at the image of this alcohol, you feel like trying it! © Paballo/31-10-15/Soul City Institute. *Source:* Photo taken by a photovoice participant—female, 21 years old, Rural Site

When reflecting on specific alcohol adverts from the media and within their local communities, it emerged strongly that youth perceive them as enticing and appealing, making them want to try the advertised beverages. Figure 7 shows a photograph taken by a young person in the rural site.

… So, you want to go for it and taste this thing and once you taste, you will end up being drunk and you want to do that forever. So, advertisements are the ones that bring us to alcohol or lead youth to alcohol (**Male**, mixed gender group, rural site).

Other elements of the adverts that attracted youth were bright colors, bold fonts, popular music, creative and enticing slogans and appealing images, scenery or settings such as the beach and ice to refresh.

**Female 1**: “People who sell alcohol, they make alcohol to seem like a cool thing… look at their facial expression as well, you know it seems like this thing makes them happy… another thing is that people advertising alcohol, make these cool slogans… **Male**:…Attractive! **Female 1**: Ja, they make them in bold letters and the important message is put in small letters…” (Mixed gender group, rural area).

Youth also indicated that alcohol adverts play on young people’s aspirations for the future. Advertisements often linked alcohol to ambition or success.

**Male 1:** “Ooh well. Yah! Alright yah, it means like, it means if you drink this one, like you dream big. Like you have that mindset of, your mind like it starts to open up to see bigger things. Yah you have a bigger picture in your mind after you have drank. **Male 2:** And you would say again like ‘Brewed for the dreamers” If you have dreams and then it gives you the courage to take action towards achieving your dreams. You see neh!” (Mixed gender group, urban site).

Participants highlighted the gendered nature of alcohol marketing. Sweet and colorful Alco-pops (flavored alcohol) and cider were seen to target young women by using attractive women in advertisements. On the other hand, men were targeted through beer adverts using symbols portraying conventional notions of masculinity such as strength, success and aspiration. Often colors reflected the target audience with softer and brighter colors for women and darker colors for men. Finally, youth in our study readily pointed out that these adverts were blatantly misleading. Based on their own observations and experiences, drinking was not always a fun and rewarding experience.

**Male 1**: “They are not showing the bad effects of alcohol, also… and obviously there will be fights, there will be one night stands after everything…**Male 2**: They are not showing the consequences…they are only showing the positive part that with alcohol there will be fun… And they don’t tell you…**Female 1**:… what happens after the fun. **Male 2:** …they don’t show you what happens after the fun ends.” (Mixed gender group, rural site).

#### Alcohol, High-Risk Sexual Behavior and Sexual Assault

**Fig. 8:**
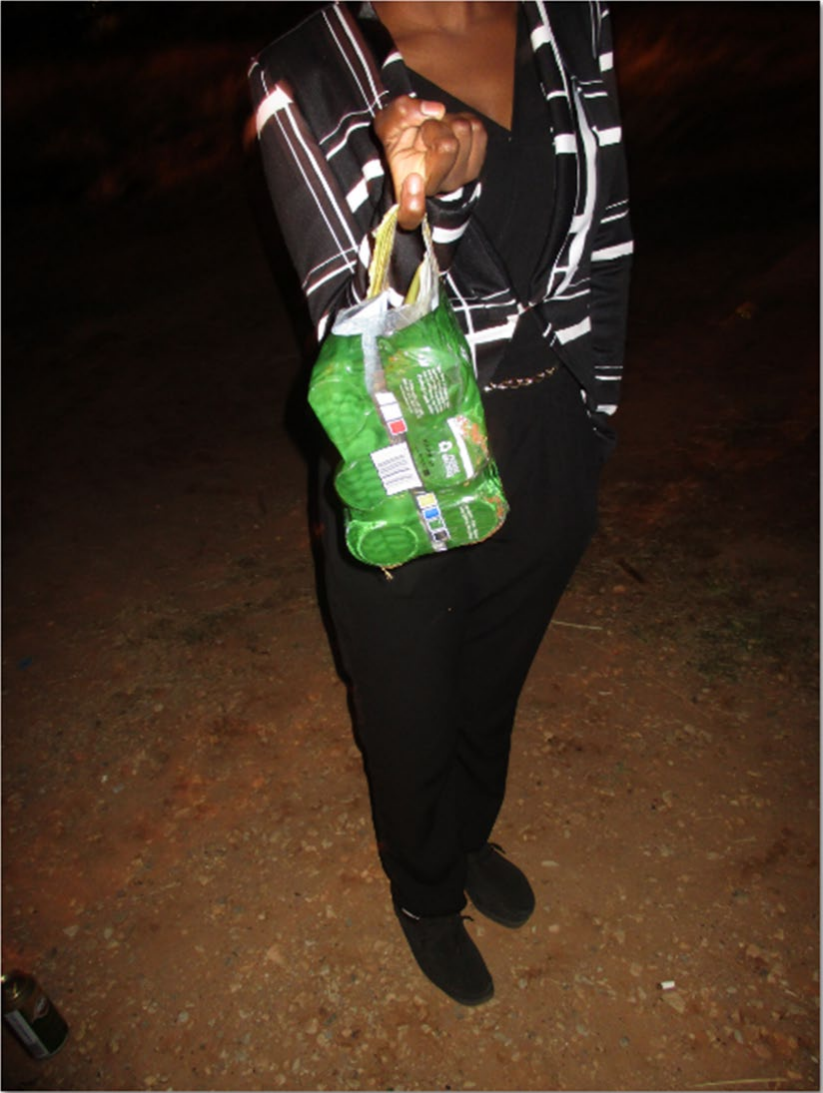
Drinking alcohol at night in the tavern alone is not safe, more especially if you are a girl like this one, because you drink too much alcohol telling yourself that you are having fun but when you are drunk, walking alone from the tavern to home you can be raped. © Kid Boy/31-10-15/Soul City Institute. *Source:* Photo taken by a photovoice participant—male, 19 years old, Rural Site

The youth who participated in this study in both urban and rural areas clearly identified the link between alcohol consumption and risky sexual behavior. They indicated that alcohol commonly led to dis-inhibition, sex that was later regretted and unprotected sex. They also reported poor decision making after consuming alcohol, which included decisions about who they go home with and the decision to have sex. Sexual dis-inhibition after drinking at taverns was reported as a common experience by young women who said they would have made different choices had they not consumed alcohol and sometimes later experienced confusion as to the consensual nature of the sexual encounter. Having unprotected sex when drunk was also common since using condoms was often overlooked, sometimes due to a diminished ability to negotiate condom use when intoxicated. Figure 8 shows a photograph taken by a young person in the rural site.

**Female:** “Some crave [for sex] and they call you to come [to a tavern]… and [say] I will buy you alcohol… and then at the end you end up not knowing what you are doing… He takes you and leaves [the tavern] with you…” (Female group, rural site).

**Male:** “…and these guys also decided to buy them [alcohol] as well. These guys like to take advantage when these girls are drunk. And these girls are young. And I don’t think that when the tavern closes they will want to go or walk home alone, just the two of them. Obviously they will offer to accompany them and that will be the start of things.” (Male group, rural site).

Alcohol consumption and drinking spaces were identified at both sites as being linked to transactional sexual relationships that could place young women at increased sexual risk. When alcohol was bought for a woman at a tavern, there was an implicit message that she would have sex with the person who bought the alcohol. Exchanging alcohol for sex was considered an acceptable transaction in the tavern setting.

**Female:** “Like you get used to drinking [over the] weekend, for you Friday, Saturday and Sunday you are on it [**All**: Yah] you don’t want to be home and just want to go out… because you can’t afford to buy alcohol every weekend, these are some of the things that make people to go to Sugar Daddys… then… they want you to sleep with them…” (Female group, urban site).

**Male:** “Yah and you tend to think that because you bought her alcohol. Now she owes you. So it becomes a problem.” (Male group, urban site).

Young women said that once the exchange of alcohol had occurred there were few options available to her if she did not want to have sex with the buyer of the drink. If she ‘ran away’ from the tavern, there was a strong belief that she would be sexually assaulted by the man at a later time. Paying back the drinks money was not an option since she was unlikely to have enough money to adequately reimburse the buyer for the drinks purchased. She would then feel compelled to have sex even if unwillingly.

**Female 1**: “you don’t even have money to go back home and he will be telling you that I am leaving you to cover the bill. What are you going to pay that with and he tells you let us just go and just do it, it will be quick and we will be done. **Female 2:** And then you end up leaving with him like that because you don’t have the money. **Female 1 interrupts:** Yah obvious… because you have to pay him off …you have to!” (Female group, urban site).

**Fig. 9:**
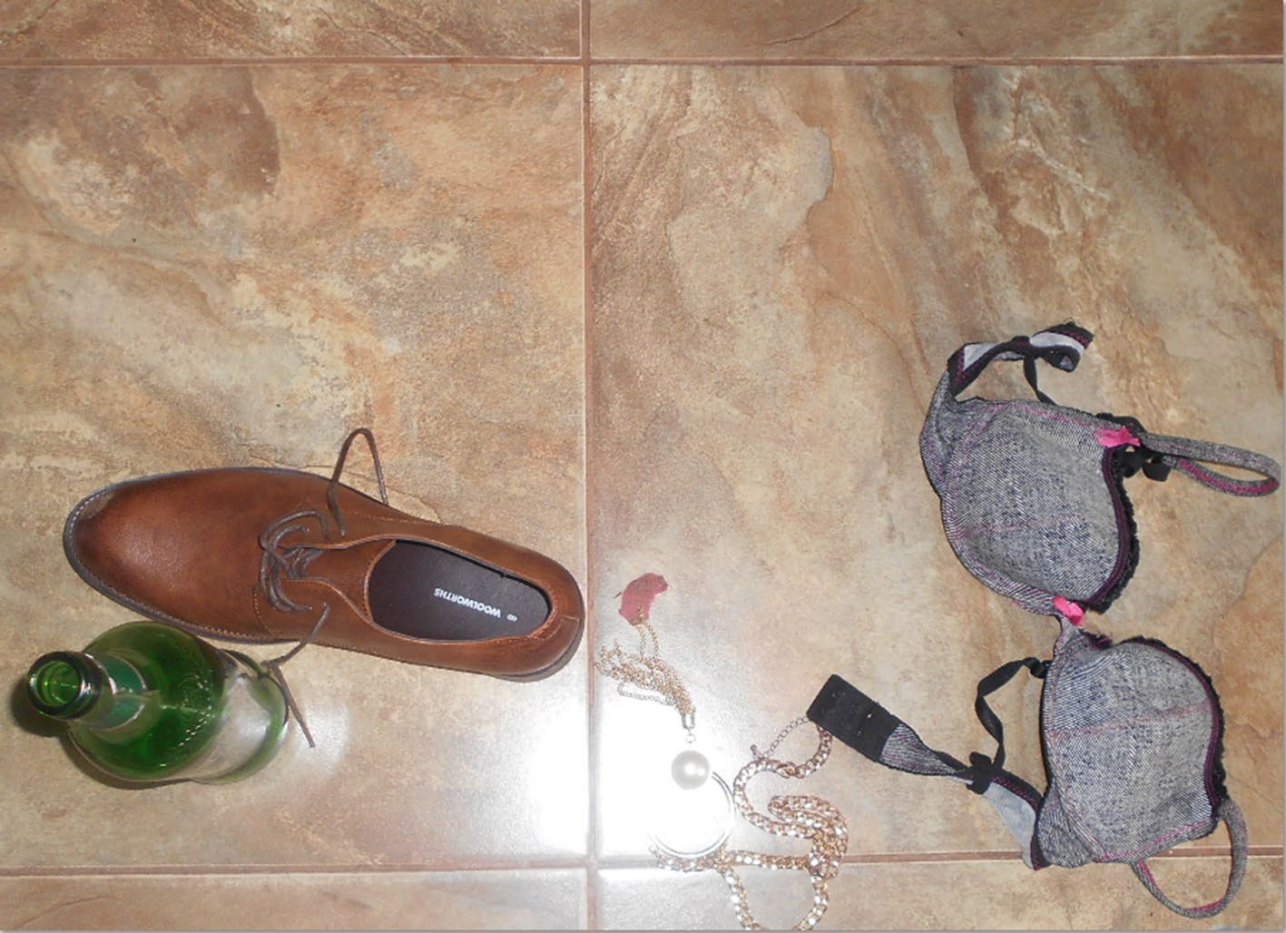
You [alcohol] be not proud. You have controlled our forefathers (ancestors) and you have controlled our parents. Now you want to control our beautiful future? You be not proud…. You destroy souls because you are a danger to everything—crime, rape, divorce, failure, accidents. You’re all about bad habits. You be not proud, you have taken lives. Most people call you refreshing but I call you a disease. You be not proud… © Tshegofatso/11-12-15/Soul City Institute. *Source:* Photo taken by a photovoice participant—female, 20 years old, Urban Site

Youth reported that drinking in taverns was associated with a greater risk of sexual assault particularly for women. This risk was believed to be from the vulnerability of a woman who is drunk and hence less able to assert herself as well as from the risk associated with walking to and from the tavern, particularly in the rural site, where transport was scarce and desolate, and open spaces were common. Figure 9 shows a photograph taken by a young person in the urban site.

**Female 1:** “… when they realize that you want to run, they are going to kick you and if they get you they are going to rape you … **Female 2**: when you are intoxicated, you are unable to fight him back. They say alcohol hits you in the knees and you are not able to fight him off.” (Female group, urban site).

**Female**: “Yes. So some end up getting killed… like you will find that taverns are far or the one where everything is happening [entertainment] is far. People leave this side to go over that side… when they pass here, like you see there are no people here it’s just an empty field, there is no one and nothing… And then you find that bad things happen, some get killed, some get raped…” (Female group, rural site).

#### Benefits of Alcohol

Participants cited various benefits of alcohol. These were income for recycling alcohol bottles, alcohol sponsorships, job creation, and social elements of having fun as well as providing a sense of confidence and bravery. However several felt that there are no real benefits to alcohol.

**Recycling**

**Male**: “I like the way he did the bottles. He is my neighbour this one… He took them and did something beautiful. He decided to recycle the bottles and decorated his yard.” (Male group, rural site).

**Sponsorship**

**Male:** “It’s having a positive impact but here, the… They act as sponsors to the teams that were playing… They just acted as sponsors, there’s nothing wrong… I don’t see any mistake here…” (Mixed gender group, rural site).

**Sales from trading**

**Female 1**: “Maybe one of those who sell alcohol. I am sure they benefit from the money they make [talking at the same time]. **Female 2**: Yah, it is them who benefit.” (Female group, urban site).

**Social confidence**

**Male**: “Some people when they drink alcohol, they say it removes shyness… S/he will think that if they drank then they will not be afraid of anyone, and that’s the benefit because they go there and are brave and not scared of anyone.” (Male group, rural site).

## Discussion

This study of alcohol, youth and sexual health risk provides insight into the community context within which youth drinking takes place in rural and urban communities in South Africa. The results of the study provide substantial evidence that young people have easy access to alcohol in their communities. Facilitated by a combination of the high density of alcohol outlets, close proximity of outlets to schools and unrestricted entry to alcohol serving venues, this availability fuels early initiation and unsafe drinking and is in conflict with both WHO recommendations and South African legislation.

The South African 2003 Liquor Act and national liquor norms and standards stipulate that alcohol should not be sold to minors or children under the age of 18 years and that alcohol outlets should verify the age of its patrons, especially youth to ensure no sales to minors [30]. The proposed liquor laws stipulate that “liquor premises be located at least five hundred meters (500 m) away from schools, places of worship, recreational facilities residential areas and public institutions” [31]. Local and community-level advocacy is required to ensure compliance, including community-based monitoring processes. The study has shown how the environment influences youth drinking which commonly occurs below 18 years of age, and often involves binge drinking and forms a key element of youth recreation.

This study further highlights the efforts by the alcohol industry to reach youth through product formulation, advertising and other types of promotion. Young adults constitute a large proportion of the population in countries such as South Africa and are a lucrative potential market. A study conducted by the South African Medical Research Council in the Tshwane district has shown that young people are heavily exposed to alcohol marketing [32]. The WHO-sponsored study titled “Monitoring Alcohol Marketing Practices in Africa” (MAMPA) assessed alcohol marketing in four African countries and concluded that given the large volume of advertising on multiple platforms carrying strong themes of success and aspiration, alcohol advertising self-regulation which is already supposed to be in place is not effective [33].

Our study found that young women are specifically targeted through marketing approaches that include images of sophistication, happiness and attractiveness. This reflects an international trend where alcohol producers are increasingly targeting women by introducing new products designed for women as well as advertising and packaging that are appealing to women [34].The use of youth-friendly media, especially social media and music, further draw youth to alcohol products. This highlights the need for public education and media literacy to challenge the existing norms that advertisers use to attract youth to consume alcohol.

Our results further highlight the link between alcohol consumption and behaviors that increase the risk of HIV and sexual assault. Youth link alcohol consumption, which commonly occurs at taverns, to unplanned, unprotected and regretted sex, and sexual assault. This supports existing evidence by other researchers that suggest that there are a set of interlinking gender and drinking norms that support the processes that lead up to sexual risk associated with drinking [1, 9, 13]. Drinking spaces, particularly taverns, are perceived to be sites for both drinking in high volumes and meeting people with whom a sexual encounter is likely to take place that same evening [14–18]. Access to alcohol for young women is heightened through men purchasing them drinks, with an expectation of sex in return for the alcohol. Where drinks are bought by men, and accepted by women, there can be an expectation that the interaction will culminate in sex, with women giving up their right to refuse. The consequences for not consenting to sex could be severe, including rape [35].

An urban and a rural community were chosen to allow for comparison purposes in terms of the influence of alcohol as a structural driver for HIV and safety. Young people in both the rural and urban area generally perceived the issues of alcohol availability and marketing as a big problem in their communities. The differences were noted in the methods of promotional activities in communities, with billboards only found in the urban area while celebrity figures were often used for events in taverns in the rural area which resulted in youth attendance as that was the only opportunity for them to see the celebrities.

All alcohol outlets were within a 500 m radius to schools in the urban area while 79% of the outlets were within 500 m in the rural area. HIV prevention efforts addressing the risks of alcohol use need to also be tailored to alcohol drinking or selling places and where young people commonly gather to have entertainment including recreational parks “youth hang out spots”.

Poor lighting in the rural area was highlighted as a risk for young women to be sexually assaulted when going home while intoxicated from taverns. There appear to be several pathways leading to alcohol-related sexual assault. In addition to possible rape consequent to not consenting to sex after accepting drinks, rape while walking from a tavern as well as ‘incapacitated’ rape where the woman was too drunk to resist sex and/or was not sure if she had consented, were described. The link between alcohol consumption amongst women and sexual assault has been previously documented. Testa and Livingstone in a literature review concluded that drinking, particularly heavy episodic drinking, plays a significant role in a large proportion of sexual assault incidences amongst young women [36]. In South Africa, a study in the Eastern Cape found that alcohol was an important part of the context for gang rapes [37]. Male perpetrators also reported that women, at the time of being raped, were sometimes too drunk to stop them [38].

Key to a public health response to alcohol-related sexual risk and HIV prevention is the creation of safer drinking spaces, which has been advocated since 2010 by the Soul City Institute through the Phuza Wize campaign. The Phuza Wize campaign aimed to encourage safe drinking and reduce alcohol-fueled violence through training communities to create safer social drinking spaces using the 10 point criteria as recommended by the WHO. The campaign also had an advocacy focus that sought to influence policy formulation, government prioritization, and public discourse to protect communities from alcohol-related harm and violence in South Africa.

In South Africa, regulatory provisions could facilitate this through ensuring limited opening hours of alcohol outlets, separate toilets for men and women and provision of condoms at taverns [30]. Resources need to be allocated efficiently to ensure both increased public and tavern owner awareness of these provisions, coupled with training and support for their implementation. Promising interventions located at drinking venues need further evaluation and scale up. For example, Morojele and colleagues found that a peer-led safe sex program including conveying safe sex messages piloted in South Africa was well received and supported by bar patrons and servers while a brief community-based intervention including skills training for sexual negotiation and condom use led to safer sexual behaviors including a decrease in partners who met at a drinking establishment [14].

The following limitations apply to this study:

- Only information observable on the exterior of the outlets were collected through the GIS mapping process, therefore, only licensed outlets were mapped. Alcohol sales in unlicensed establishments is common in South African communities, which are also frequented by youth and adults [8, 30, 39, 40]. Excluding venues that were unlicensed means that the density of alcohol establishments was underestimated in this study.
- Convenience sampling was used to recruit youth either through local CBOs or a Soul City Institute implemented HIV-prevention program, which is likely to have led to a biased sample since participants may have been sensitized to social issues through these affiliations.

Despite the limitations, this study was the first in South Africa to explore, in depth young people’s perceptions and experiences of alcohol marketing and sexual health risk in their communities. It was also the first to use innovative methods such as GIS and photovoice to provide visual evidence of the high density of alcohol outlets and advertising linked to youth drinking patterns and sexual health risk. The use of photovoice also allowed youth to creatively use photography to tell their story and question the status quo.

This study of alcohol availability, alcohol marketing, drinking patterns and sexual health risk amongst South African youth has highlighted how the environments that both rural and urban youth live in are characterized by easy and widespread alcohol availability, and a bombardment of alcohol-promoting messages through marketing and advertising. By young people’s own estimation, these factors in turn influence drinking patterns, resulting in substantial risk for youth, particularly sexual risk and violence for young women. Our results underline the importance of developing and implementing strategies at policy and community levels that tackle the broader context and structural factors to protect youth. The influence of alcohol availability and advertising on youth drinking patterns needs to be countered by a strengthened legislative framework complemented by strong implementation of both existing regulations and evidence-based interventions at community level. Alcohol as a structural driver of HIV and sexual health risk requires strong structural solutions that address policies and implementation from national to community levels. It is imperative that the proposed South African alcohol legislature that seeks to safeguard young people from high exposure to alcohol availability and marketing, be passed into law in order to protect them from harmful use of alcohol and associated sexual risk including HIV and sexual violence.
